# Supplementary figures and images for: Molecular epidemiology of carbapenem-resistant Escherichia coli in a tertiary hospital located in the Dabie Mountains region, China
Source: PeerJ. 2025 Oct 24;13:e20188. doi: 10.7717/peerj.20188 (PMC12558158; doi:10.7717/peerj.20188)

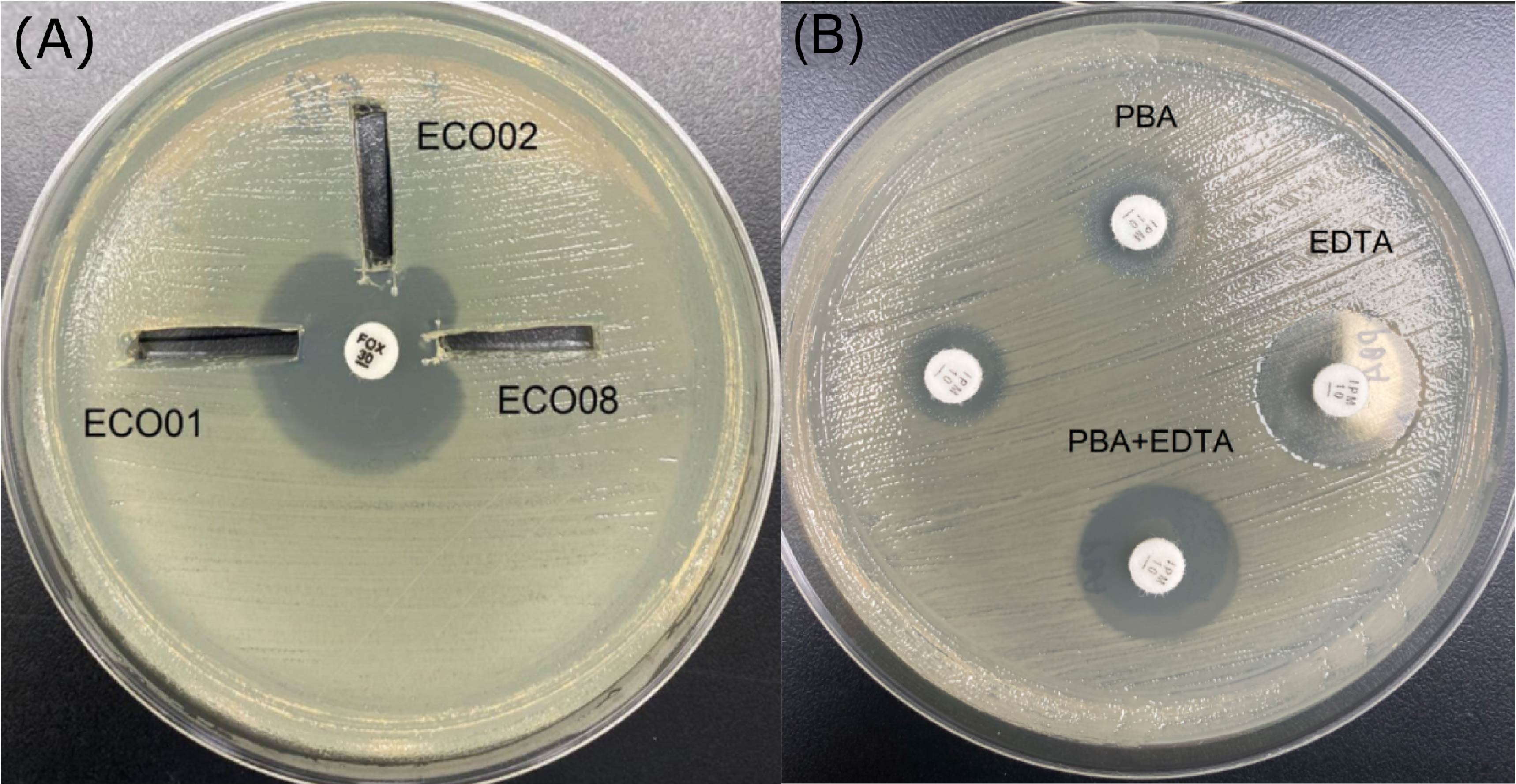

Supplement: Supplemental Information 1 — (A) Enhanced growth of the surface organism (E. coil ATCC 25922), was seen near agar slits (arrows) that contain extracts of ECO02 and ECO08 test isolates, both of which are AmpC producer; (B) The difference in zone size in the presence and absence of EDTA was ≥5mm for IPM, suggesting MBL production. EDTA: ethylenediaminetetraacetic acid; PBA: phenyl boronic acid; IPM: imipenem; MBL: metallo-β-lactamase. [file peerj-13-20188-s001.jpg]

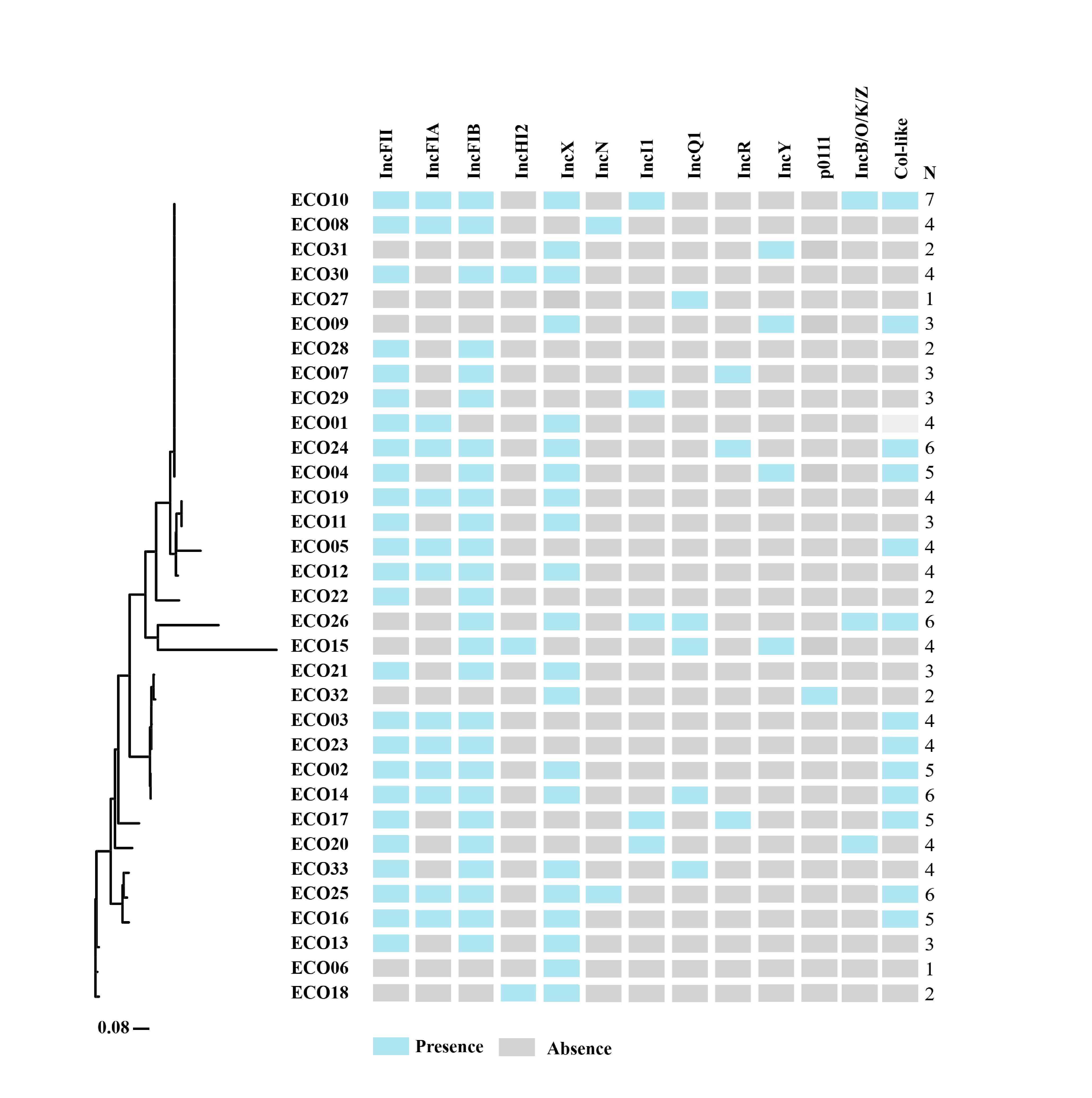

Supplement: Supplemental Information 3 [file peerj-13-20188-s003.jpg]
